# Determinants of Hurricane Evacuation from a Large Representative Sample of the U.S. Gulf Coast

**DOI:** 10.3390/ijerph16214268

**Published:** 2019-11-03

**Authors:** Ibraheem M. Karaye, Jennifer A. Horney, David P. Retchless, Ashley D. Ross

**Affiliations:** 1 Program in Epidemiology, University of Delaware, Newark, DE 19716, USA; horney@udel.edu; 2 Department of Marine Sciences, Texas A&M University at Galveston, Galveston, TX 77554, USA; retchled@tamug.edu (D.P.R.); ashleydross@tamug.edu (A.D.R.)

**Keywords:** hurricane, evacuation, U.S. Gulf Coast, disaster, vulnerability

## Abstract

Exposure to natural disasters like hurricanes negatively impacts the mental and physical health of populations, and evacuation is an important step taken to prevent these adverse health events. This study uses data from a large representative sample of U.S. Gulf Coast residents to explore the determinants of hurricane evacuation. In December 2017, data were collected from 3030 residents of the U.S. Gulf Coast, including Texas, Louisiana, Mississippi, Alabama, and Florida—2557 of whom reported being impacted during the 2017 hurricane season. Bivariate analyses were conducted using prevalence differences and tested for statistical significance with chi-square tests. Multivariable logistic regression models were fitted to identify factors associated with hurricane evacuation. One-third of the respondents (919 of 2557; 35.9%) evacuated from a hurricane that impacted the U.S. Gulf Coast in 2017. The determinants of hurricane evacuation in this population were: residing in a mobile home, higher perception of storm surge risk, higher perception of wind risk, self-sufficiency, carrying flood insurance, and reliance on media and family for evacuation decisions. These findings may be relevant for reducing the adverse health effects of hurricanes by improving emergency planning and evacuation in this highly vulnerable region.

## 1. Introduction

Hurricanes can have a devastating toll on an exposed population’s health due to the direct and indirect effects of flooding, storm surge, tornadoes, and damaging winds [1,2]. For example, physical health outcomes like injuries, infections, and deaths have been recorded following hurricanes, due to water-borne outbreaks, drowning, transmission of mosquito-borne diseases, environmental pollution, and the disruption of basic health care services during hurricane conditions [3,4,5]. The rate of mental health illnesses such as post-traumatic stress disorder (PTSD), major depressive disorder, acute stress disorder, and generalized anxiety disorder has also been shown to increase following hurricane exposure [2,3,6,7].

Evacuation is an important strategy that has been shown to reduce adverse health outcomes associated with disasters [8,9]. However, even with the known health benefits associated with evacuation as an emergency management intervention, some residents do not evacuate in the face of a landfalling hurricane [10]. Determining why people fail to evacuate from an impending disaster has been difficult [11,12,13]. Predicting evacuation requires assessment of individual decision-making. One model known as the Protective Action Decision Model (PADM) suggests that when individuals are faced with disaster, they make evacuation decisions based on environmental cues, social cues, personal experiences, and evacuation impediments [14]. Environmental cues include geographic or structural location of the individuals, such as risk area (e.g., proximity to water bodies) and mobile home residence. Social cues involve the observation of peers, family or neighbors evacuating and the issuance of evacuation orders by authorities, while evacuation impediments entail concerns about property damage, theft, scarcity of resources to finance evacuation, and traffic on evacuation routes. A relatively large body of research has tested associations between these factors, and others, across storms of various strength (e.g., Category 1–5 on the Saffir Simpson scale), in different locations (e.g., U.S. Atlantic of Gulf Coast), and at different time points [9,12,13,15].

Overall, the determinants of hurricane evacuation have been studied widely but the results have remained largely inconsistent since the first review was published in 1980 [16]. Although many factors might explain this variability, several studies have proposed that region of residence may be a potential modifier of the reported associations with evacuation [17,18]. Unfortunately, a majority of the previously published studies on hurricane evacuation were not designed to capture the effects of moderating variables, reporting only population-average risks that convey little information about sub-populations. This study uses primary data collected from a representative sample of U.S. Gulf Coast residents following the 2017 North Atlantic hurricane season to test correlations between evacuation behavior and environmental and social cues, among other factors. The 2017 season was the costliest on record, with 17 named storms and three major hurricanes: Harvey, Irma, and Maria [19]. Thus, the main aim of this study is to identify factors that predict hurricane evacuation from a large representative sample of the U.S. Gulf Coast.

### 1.1. Background

#### 1.1.1. Environmental and Social Cues

The association between environmental cues in the form of mobile home residence and risky geographic location are well documented in the literature. Studies have found residents of mobile homes are more likely to evacuate an impending storm [20,21,22,23]. Mobile home residents likely perceive their homes as not sturdy enough to withstand the impact of hurricanes, which heightens their risk perception, and hence the decision to evacuate [10]. Similar to mobile home residence, living in a high risk area, such as near water, has also been shown to be associated with hurricane evacuation, as individuals are often conscious of their proximity to the coast and increased likelihood of being impacted by a hurricane’s storm surge or associated flooding [10,17,18].

Social cues entail information gathered from formal and informal sources. The literature on the association between dissemination of information and evacuation of residents exposed to hurricanes has been mixed. While some studies report that non-media sources like family, friends, and the government are associated with hurricane evacuation [24,25], others have shown that the media exert a more significant influence on hurricane evacuation decision [17,26].

#### 1.1.2. Perception of Risk

Residents are more likely to evacuate if they have a higher perception of the potential risks, such as the risk of injury or the risk of property damage [11,27]. Perception of wind risk has been shown to be consistently associated with evacuation, while perception of flood or storm surge risk have had mixed effects on evacuation [10,27].

#### 1.1.3. Socioeconomic Factors

Demographic factors like race, age, and income generally represent socioeconomic vulnerabilities and as such are important to consider in the context of evacuation; however, these factors have not consistently predicted hurricane evacuation [10,11,28]. While some studies report a positive association [26,29,30], others report a negative or null association between demographic characteristics and hurricane evacuation [10,11,17,20]. For example, Peacock et al. [24] found mixed associations between race and evacuation decisions among respondents exposed to Hurricane Andrew, a Category 5 storm that made landfall in Florida in 1992. Analyses indicated that the exclusion of risk indicators in the model produced results showing that Hispanics and African Americans were less likely to evacuate than whites [24]. On the contrary, the inclusion of risk indicators in the model showed a null association between race/ethnicity and evacuation [24].

Similar to race, the reported associations between income and evacuation have been mixed, with some authors reporting a positive relationship [24,29,30,31] and others, an inverse association [20,21,32,33,34]. A positive relationship has been explained by the ability of the wealthy to finance expenses associated with evacuation, while a negative relationship may be due to calculations by wealthy residents that their homes are able to weather a storm’s impacts. The wealthy may also own a higher risk coastal property subject to waves and storm surge that leads them to remain to protect their homes from damage. 

In an attempt to explain the inconsistency between demographic factors and evacuation, Horney [28] collected data from 90 census blocks in three eastern North Carolina counties affected by Hurricane Isabel and found that social factors (e.g., social capital, social cohesion, social control) modified the association between evacuation and demographic variables. For example, analyses indicated that race had a differential effect on evacuation depending upon respondents’ social cohesion. Nonwhite respondents with more local friends and family were more likely to evacuate while this had no effect on the evacuation of white respondents.

## 2. Materials and Methods

### 2.1. Study Population and Data Collection

Study participants were recruited from the geographic area consisting of the 73 National Oceanic and Atmospheric Administration’s (NOAA) designated Coastal Zone Management Program (CZMP) counties in Texas, Louisiana, Mississippi, Alabama, and Florida. The survey was conducted from 11 December 2017 through 11 January 2018. The survey panel met quotas to replicate population proportions for age (18–24 years 12.48%; 25–44 years 34.73%; 45–64 years 34.30%; 65 years and older 18.49%), sex (female—51.36%; male—48.64%), race/ethnicity (white—55.61%; Hispanic/Latino—23.27%; African American—16.39%; other—4.73%), and state of residence (Alabama—3.85%; Florida—39.98%; Louisiana—13.65%; Mississippi—2.4%; Texas—40.12%), using U.S. Census Bureau data [35]. Using a cross sectional study design, the survey was administered online to a panel of 3030 participants by Qualtrics International Inc. (Provo, UT, USA). Participants were asked questions about their demographic characteristics, risk perceptions, and evacuation decisions during Hurricanes Harvey, Maria, Irma, and Nate. A copy of the survey instrument is available as a supplemental attachment (see Supplemental File). Human subjects’ research approval of this study was obtained from the Institutional Review Board of Texas A&M University at Galveston (IRB2017-0916M).

### 2.2. Statistical Analysis

Descriptive statistics including, frequencies, counts, and 95% confidence intervals (CI) were calculated using Stata 15.1 (College Station, TX, USA). Crude prevalence differences and 95% CIs were estimated using the *csi* command and hypotheses tested with chi-square tests. A multivariable logistic regression model was then fitted with hurricane evacuation (yes, no) as the outcome variable and home type (single family home, mobile home, apartment, other), perception of surge risk (low, medium, high), perception of flood risk (low, medium, high), perception of wind risk (low, medium, high), influence of neighbor on evacuation decision (yes, no), influence of local authorities on evacuation decision (yes, no), influence of family on evacuation decisions (yes, no), self-sufficiency for disaster recovery (yes, no), hurricane impact on property (0–100), and home flood insurance (yes, no) as predictor variables. Reference categories were chosen because they were previously identified in the literature or assumed by the study authors to be the lowest risk level. The model was adjusted for age (18–24 years; 25–44 years; 45–64 years; 65 years and older), sex (male, female), and race/ethnicity (white, Hispanic/Latino, African American/other) of the respondents by applying a survey weight using the *svyset* command to all analyses.

## 3. Results

A total of 2557 of 3030 respondents reported exposure to a hurricane in the U.S. Gulf Coast in 2017. The mean (SD) age of the respondents was 46.12 (17.20) years old and the sample was 51.4% female (*n* = 1314) and 48.6% male (*n* = 1243). One-third of the respondents (919 of 2557; 35.9%) reported evacuating due to a hurricane in the U.S. Gulf Coast in 2017, while 64.1% remained in their homes. Overall, 73.0% (*n* = 1866) of the respondents reported living in single family homes as opposed to mobile homes or apartments, and 65.5% were homeowners (1674 of 2557) rather than renters. Self-sufficiency was defined as an affirmative response to the question, “Should individuals bear the cost of recovery from a natural disaster on their own (rather than the government)?” Self-sufficiency is also defined by the Federal Emergency Management Agency (FEMA) as being able to live without running water, electricity/or gas, and telephones for at least three days after a disaster [36]. A majority of the respondents (84.3%; *n* = 2157) reported that they would rely on the government for disaster recovery assistance, while 15.7% (*n* = 400) of the respondents reported that they were self-sufficient (Table 1).

In the bivariate analyses of evacuation and predictor variables, evacuees were more likely to be Hispanic (PD = 0.08; 95% CI: 0.03, 0.13), African American (PD = 0.07; 95% CI: 0.03, 0.12), live in mobile homes (PD = 0.25; 95% CI: 0.18, 0.32), and have a higher perception of surge or flood risk than non-evacuees. The prevalence differences can be interpreted to mean that respondents who lived in mobile homes had 25 additional evacuees per 100 compared to those who lived in single family homes, and the respondents who had high perception of surge risk had 42 additional evacuees per 100 compared to those with low perception of surge risk (Table 2).

### Predictors of Hurricane Evacuation

In a multivariable logistic regression model of evacuation on predictor variables, the odds of evacuation were found to be significantly higher among mobile home dwellers (OR = 3.31; 95% CI: 2.32, 4.71), those with higher perception of surge (OR = 4.97; 95% CI: 3.68, 6.70) or wind risk (OR = 1.56; 95% CI: 1.14, 2.14), the self-sufficient (OR = 1.53; 95% CI: 1.18, 1.99), and those who rely on media (OR = 1.86; 95% CI: 1.31, 2.66) or family for evacuation decisions (OR = 2.12; 95% CI: 1.61, 2.79). Conversely, the odds of evacuation were found to be significantly lower among respondents who reported carrying flood insurance on their homes (OR = 0.71; 95% CI: 0.60, 0.83) (Table 3).

## 4. Discussion

Evacuation is an important strategy that can be ordered by authorities for at-risk populations to reduce the adverse health effects of hurricane exposure [8,9]. Exposure to disasters or needed to be rescued by authorities or volunteers from a disaster have been associated with a higher prevalence of mental health disorders such as PTSD [3,37,38]. Identifying factors that predict evacuation from disasters may limit the occurrence of these adverse health outcomes. In this study, demographic- and storm-related factors associated with hurricane evacuation in the U.S. Gulf Coast were identified. Perception of risk was positively associated with hurricane evacuation because it likely estimates the self-rated threat of hurricanes to individuals, their families, and properties [39,40,41,42]. Because of the structural vulnerability of mobile homes, residents likely take this environmental cue to indicate a higher risk of storm damage and evacuate an impending storm [10,25].

Respondents reporting having flood insurance—the insurance coverage against property loss or damage during flooding—were less likely to have evacuated. This might be due to their low perception of risk towards the hurricane. For example, in a study conducted on Hurricane Andrew, Peacock, Gladwin, and Morrow [24] found that residents with flood insurance or stronger home structures were less likely to evacuate because they perceived lower risk of damage from the storm.

Respondents who reported self-sufficiency were more likely to evacuate than those who relied on the government for disaster recovery needs. This is expected since the self-sufficient are more likely to finance their evacuation and bear the costs of damage to their homes and properties [36]. This may reflect the emphasis on household preparedness by federal agencies; for example, individuals are urged to have enough food, water, and medicines for their family for three days in case of a disaster event and plan for financing disaster recovery [43]. Research has shown, however, that low-income minority households often lack the funds needed to be self-sufficient in this sense [44], suggesting that perceptions of self-sufficiency may intersect with race and income. Other studies of the Gulf Coast region have found that perceptions of self-sufficiency in disasters are associated with rural communities [45]. Future studies should explore these factors further.

The association of social cues and evacuation was confirmed by this study. Reliance on family for evacuation decisions was found to be positively associated with evacuation. Past research has suggested this is because residents with children are more likely to perceive higher risks and comply with evacuation messages [18,20,23,39].

The significant association between reliance on media and hurricane evacuation supports previous findings by Lindell [39] and Stein et al. [17]. However, the finding conflicts with the review by Baker [25], which showed that evacuation is not strongly associated with primary source of information about storm conditions and intensity of storm warnings. The conflicting findings among studies may be explained by variation in risk perception based on geographic area of residence. For example, a study of evacuation behavior during Hurricane Rita in 2005 has shown that residents that live in evacuation zones were more likely to rely on the media for their evacuation decisions compared to residents that live outside of these zones [17]. Given that the current study was conducted on residents of the U.S. Gulf Coast, we recommend that future studies be conducted in the U.S. Atlantic Coast to compare the findings.

This study has several important limitations. The design was cross-sectional, measuring both evacuation and other variables at the same time. Therefore, no temporal relationship could be established between hurricane evacuation and any of the predictor variables. Additionally, because the data were collected via an online survey, the study participants may differ from non-participants residing in the same region due to their access to the internet and availability to devote time to participating in the study, which might result in selection bias. However, because the survey instrument was designed to replicate the U.S. Gulf Coast population for age, sex, and race, it is less likely that selection bias impacted the results.

## 5. Conclusions

This study reported the determinants of hurricane evacuation from a large representative sample of the U.S. Gulf Coast population. Medium and high perception of surge risk, high perception of wind risk, mobile home residence, self-sufficiency, reliance on media for the decision to evacuate, reliance on family for evacuation decision, and having flood insurance were found to significantly predict the evacuation decision in this population. These findings would be relevant for emergency planning and evacuation for improved health outcomes associated with hurricane exposure in this geographic region. It is recommended that the study be replicated using a large representative sample of the U.S. Atlantic Coast to also isolate the determinants of evacuation in this population.

## Figures and Tables

**Table 1:** Descriptive Statistics of Select Variables for Study Participants.

Continuous Variables	Mean (SD)
Age in Years	46.12 (17.20)
Categorical Variables	(*n* = 2557) (%)
Evacuated	
Yes	919 (35.9)
No	1638 (64.1)
Sex	
Female	1314 (51.4)
Race/Ethnicity	
White	1418 (55.5)
Hispanic or Latino	615 (24.1)
African American/Other	523 (20.5)
Age in Groups	
18–24 years	317 (12.4)
25–44 years	913 (35.7)
45–64 years	868 (34.0)
65 years and older	458 (17.9)
Self-Sufficiency	
Yes	400 (15.7)
Home Ownership	
Yes	1674 (65.5)
Home Type	
Mobile Home/Trailer	213 (8.3)
Single family	1866 (73.0)
Apartment	478 (18.7)
Educational Status	
High School	546 (21.4)
Some College	681 (26.6)
Associate/Bachelor’s Degree	895 (35.0)
Graduate/Professional degree	435 (17.0)
Perception of Flood Risk	
Low	699 (27.4)
Medium	973 (38.0)
High	885 (34.6)
Perception of Surge Risk	
Low	1074 (42.0)
Medium	776 (30.4)
High	706 (27.6)

**Table 2:** Distribution, Crude Prevalence Differences, and 95% Confidence Intervals (95% CI) for Demographic Factors Potentially Associated with Evacuation from Hurricanes in the Gulf Coast in 2017 (*n* = 2557).

Variable Description	Evacuated		Did Not Evacuate		Prevalence Differences
	(*n* = 929)		(*n* = 1628)		(95% CI)
	*n*	%	*n*	%	
**Home Type**					
Single family	632	33.85	1235	66.15	REF
Mobile Home/Trailer	123	58.85	86	41.15	* 0.25 (0.18, 0.32)
Apartment/Other	174	36.17	307	63.83	0.02 (−0.02, 0.07)
**Sex**					
Female	513	38.08	834	61.92	REF
Male	416	34.38	794	65.62	−0.04 (−0.07, 0.00)
**Age**					
18–24 years	137	40.53	201	59.47	REF
25–44 years	452	44.53	563	55.47	0.04 (−0.02, 0.10)
45–64 years	184	30.16	426	69.84	* −0.10 (−0.17, −0.04)
65 years and older	156	26.26	438	73.74	* −0.14 (−0.21, −0.08)
**Race/Ethnicity**					
White	452	32.78	927	67.22	REF
Hispanic or Latino	256	40.76	372	59.24	* 0.08 (0.03, 0.13)
African American/Other	221	40.18	329	59.82	* 0.07 (0.03, 0.12)
**Educational Status**					
High school	218	40	327	60	REF
Some college	225	32.99	457	67.01	* −0.07 (−0.12, −0.16)
Associate/Bachelor’s degree	306	34.54	580	65.46	* −0.05 (−0.11, −0.00)
Graduate/Professional degree	180	40.54	264	59.46	0.01 (−0.06, 0.07)
**Perception of Flood Risk**					
Low	156	22	553	78	REF
Medium	341	35.82	611	64.18	* 0.14 (0.10, 0.18)
High	432	48.21	464	51.79	* 0.26 (0.22, 0.31)
**Perception of Surge Risk**					
Low	186	17.46	879	82.54	REF
Medium	315	40.54	462	59.46	* 0.23 (0.19, 0.27)
High	428	59.86	287	40.14	* 0.42 (0.38, 0.47)

* *p*-value < 0.05. REF = referent group.

**Table 3:** Predictors of Hurricane Evacuation in the U.S. Gulf Coast.

Home Type (Ref. = Single Family)	Odds Ratio	95% CI
Mobile Home/Trailer	* 3.31	2.32, 4.71
Apartment/Other	1.20	0.93, 1.53
**Surge Risk (Ref. = Low)**		
Medium	* 2.58	1.97, 3.37
High	* 4.97	3.68, 6.70
**Flood Risk (Ref. = Low)**		
Medium	0.81	0.61, 1.08
High	0.87	0.63, 1.19
**Wind Risk (Ref. = Low)**		
Medium	1.14	0.83, 1.57
High	* 1.56	1.14, 2.14
**Self Sufficient (Ref. = No)**		
Yes	* 1.53	1.18, 1.99
**Reliance on Media (Ref. = No)**		
Yes	* 1.86	1.31, 2.66
**Reliance on Family (Ref. = No)**		
Yes	* 2.12	1.61, 2.79
**Flood Insurance (Ref. = No)**		
Yes	* 0.71	0.60, 0.83
**Age (Ref. = 18–24 years)**		
25–44 years	1.25	0.93, 1.68
45–64 years	0.93	0.66, 1.31
65 years and older	1.02	0.70, 1.49
**Race/Ethnicity (Ref. = White)**		
Hispanic	0.99	0.77, 1.29
African American/Other	1.25	0.96, 1.62
**Sex (Ref. = Male)**		
Female	0.95	0.76, 1.17
**Educational Status (Ref. = High School)**		
Some College	0.89	0.67, 1.18
Associate/Bachelor’s Degree	0.90	0.68, 1.18
Graduate/Professional Degree	1.16	0.84, 1.60

* *p*-value < 0.05.

## Supplementary Materials

The following are available online at https://www.mdpi.com/1660-4601/16/21/4268/s1, survey instrument.
