# Supplementary material for: Determinants of Hurricane Evacuation from a Large Representative Sample of the U.S. Gulf Coast
Source: Int J Environ Res Public Health. 2019 Nov 3;16(21):4268. doi: 10.3390/ijerph16214268 (PMC6861906; doi:10.3390/ijerph16214268)
Supplement: Supplementary file 1 [file ijerph-16-04268-s001.pdf]

International Journal of
Environmental Research and Public Health

MDPI

SUPPLEMENTAL FILE: SURVEY INSTRUMENT

Thank you for your interest in completing our survey. This survey will ask you about your perceptions of climate change and coastal hazards as well as your experience with disasters. Participation in this survey is voluntary. To participate you must be 18 years of age or older. You must also be a resident of a coastal county or parish. You have a right to withdraw from the study at any time without consequences. The information you share is anonymous. People who have access to this information include the Principal Investigators and research study personnel.

Do you agree to participate?

Note that you may print this for your records.

- ○ Yes
- ○ No

What is your gender?

- ○ Male
- ○ Female

Please select your age range.

- ○ Less than 18 years
- ○ 18-24 years
- ○ 25-44 years
- ○ 45-64 years
- ○ 65 years and older

Which of the following do you most closely identify with?

- White
- Hispanic or Latino
- African American
- Other race/ethnic group

In which state do you currently reside?
In which county do you currently reside?

First, we would like to know your thoughts on hurricanes. For the questions that follow, "major hurricane" means one with wind speeds above 110 miles per hour or Category 3 or more.

How much risk or danger do you feel you are at from major hurricanes?

Slide the marker to indicate your feeling on a scale of 0 (none at all) to 100 (extreme).

	0 10 20 30 40 50 60 70 80 90 100
Feeling at risk to hurricanes	

Tell us how much you agree with the following statements.	Strongly disagree	Somewhat disagree	Neither agree nor disagree	Somewhat agree	Strongly agree
I'm worried about the danger of a storm surge on the Gulf Coast.	○	○	○	○	○
A storm surge can have fatal consequences for the coastal area and its inhabitants.	○	○	○	○	○
Living on the Gulf Coast is a threat to my safety.	○	○	○	○	○
I greatly expect storm surge to cause floods in coastal areas.	○	○	○	○	○
When I think of floods, I feel concerned.	○	○	○	○	○

How much harm do you think would come to you personally (to your family, property, job, etc.) if the following were to happen? Assume each hazard is of moderate intensity.

	None	A litle	Some	A Lot
Hurricane wind	○	○	○	○
Hurricane storm surge	○	○	○	○
Flooding from rainfall	○	○	○	○
Flooding from river overflow	○	○	○	○
Tornado	○	○	○	○

How much trust do you have in federal, state, and local government to protect you in a disaster event?

	None	A little	Some	A lot
Federal government	○	○	○	○
State government	○	○	○	○
Local government (your county or city)	○	○	○	○

Consider the following pair of statements. Which comes closest to your personal view?

- ○ Government has a responsibility to help people respond and recover from natural disasters.
- ○ Individuals have a responsibility to be self-sufficient when it comes to natural disasters.

Did a hurricane this year in the Gulf Coast region threaten you or your community?

- ○ Yes
- ○ No

Thinking back to the days and hours before the hurricane made landfall, how much risk or danger did you feel your home and family were at to the following?

	Low	Medium	High
Wind	○	○	○
Flood	○	○	○
Storm surge	○	○	○

Did you evacuate due to the threat of the hurricane?

- ○ Yes
- ○ No

How much of your decision to evacuate (or not) was based on information you received from...	Completely	Somewhat	No influence
The media	○	○	○
Local authorities	○	○	○
Neighbors	○	○	○

Close friends and family	○	○	○

What impact did the hurricane this year have on your home and personal property?

Slide the marker to indicate damages from 0 (none at all) to 100 (extreme devasation).

	0 10 20 30 40 50 60 70 80 90 100
Damages to home and property	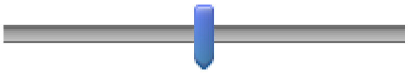

What impact did the hurricane this year have on your...?

	Very positive	Mostly positive	Some negative and some positive	Mostly negative	Very negative	No impact
Job	○	○	○	○	○	○
Household finances	○	○	○	○	○	○
Physical health	○	○	○	○	○	○
Mental and emotional health	○	○	○	○	○	○

What kind of home do you live in?

- ○ Mobile home or trailer
- ○ Single-family home, not attached to another house
- ○ Single-family home, attached such as a duplex/quadriplex
- ○ Apartment in an apartment building
- ○ Other (please specify): ____________________

Do you own or rent your home?

- ○ Own
- ○ Rent

Do you carry the following insurance policies on your home?

	Yes	No	I don't know.
Flood insurance	○	○	○
Wind insurance	○	○	○
Content insurance	○	○	○

Generally speaking, would you say that most people can be trusted or that you can't be too careful in dealing with people?

- ◯ Can trust
- ◯ Cannot trust
- ◯ Depends

What is your age (in years)?

▼ 18 ... 100

Which race do you most closely identify with?

- ◯ White or Caucasian
- ◯ African-American
- ◯ Asian-American
- ◯ American-Indian
- ◯ Pacific-Islander American
- ◯ Mixed Race
- ◯ Other ________________________________
